# Curcumin and Dementia: A Systematic Review of Its Effects on Oxidative Stress and Cognitive Outcomes in Animal Models

**DOI:** 10.3390/ijms26147026

**Published:** 2025-07-21

**Authors:** Samuel Abiodun Kehinde, Wai Phyo Lin, Bo Bo Lay, Khin Yadanar Phyo, Myat Mon San, Rinrada Pattanayaiying, Sasitorn Chusri

**Affiliations:** 1 Biomedical Technology Research Group for Vulnerable Populations and School of Health Science, Mae Fah Luang University, Muang, Chiang Rai 57100, Thailand; samuelabiodun.research@mfu.ac.th (S.A.K.); 6431804208@lamduan.mfu.ac.th (W.P.L.); 6531804115@lamduan.mfu.ac.th (K.Y.P.); 6531804120@lamduan.mfu.ac.th (M.M.S.); 2 Biochemical/EnTox Lab., Faculty of Basic Medical Sciences, Ajayi Crowther University, Oyo 211001, Nigeria; 3 School of Information Technology, Mae Fah Luang University, Muang, Chiang Rai 57100, Thailand; 6431306113@lamduan.mfu.ac.th; 4 Department of Food Innovation and Professional Chef, Faculty of Science and Technology, Suan Sunandha Rajabhat University, Bangkok 10300, Thailand; rinrada.pa@ssru.ac.th

**Keywords:** curcumin, curcuma longa, dementia, oxidative stress, neuroinflammation, antioxidant, cognitive function, animal models

## Abstract

Dementia is marked by progressive cognitive decline linked to oxidative stress, neuroinflammation, and synaptic dysfunction. Curcumin, a natural compound from Curcuma longa, has shown promising neuroprotective effects. This systematic review analyzed 29 preclinical studies using rodent models of dementia induced by chemical, genetic, or dietary methods. The review focused on curcumin’s effects on oxidative stress, inflammation, and cognitive outcomes. All studies assessing malondialdehyde (MDA) reported significant reductions, indicating reduced oxidative stress. Superoxide dismutase (SOD) activity increased in all measured cases, while glutathione (GSH) levels rose in about one-third of studies. A literature search was comprehensively conducted using PubMed, Scopus, AMED, and LILACS databases through April 2024. Curcumin also demonstrated anti-inflammatory properties, with over 80% of studies showing reduced levels of pro-inflammatory cytokines such as TNF-α, IL-6, and IL-1β. Additionally, 40% of studies noted increases in anti-inflammatory markers like IL-4 and IGF-1. Cognitive performance improved in around 80% of studies, especially in spatial learning and memory. Some studies also reported behavioral improvements, including reduced anxiety and enhanced locomotion. Curcumin demonstrated potent antioxidative, anti-inflammatory, and cognitive-enhancing effects across diverse dementia models. Its ability to modulate multiple pathological pathways highlights its potential as a bioactive compound for mitigating cognitive decline associated with neurodegenerative diseases. However, variability in study design and curcumin formulations suggests the need for standardized protocols and further high-quality research to facilitate clinical translation.

## 1. Introduction

Dementia refers to a group of progressive neurodegenerative disorders marked by cognitive decline, memory loss, impaired reasoning, and behavioral changes [1,2]. It poses a growing global health burden, with over 55 million people affected and nearly 10 million new cases annually [3,4,5]. Alzheimer’s disease (AD) is the most prevalent type, comprising 60–70% of reported cases, followed by vascular dementia, Lewy body dementia, and frontotemporal dementia. As populations age, dementia prevalence is expected to rise dramatically, reaching 139 million by 2050. Economically, the condition is highly burdensome, with global costs exceeding USD 1.3 trillion in 2019 and projected to double by 2030 [6].

Oxidative stress is a major pathological hallmark of dementia and significantly contributes to the initiation and progression of neurodegenerative processes [7,8]. It arises when the production of reactive oxygen species (ROS) exceeds the capacity of the body’s antioxidant defenses. The brain is particularly vulnerable to oxidative injury due to its high rate of oxygen utilization, abundance of lipids, and relatively low antioxidant capacity. This imbalance results in damaging effects such as lipid peroxidation, protein and DNA oxidation, and mitochondrial impairment, ultimately leading to neuronal loss and cognitive deterioration [9,10].

Accurate and early diagnosis of dementia is crucial for effective disease management and for assessing therapeutic strategies in both clinical and research settings. While traditionally based on clinical evaluation using standardized criteria, dementia diagnosis has increasingly incorporated biomarkers that reflect underlying pathophysiological processes. Three major diagnostic frameworks are widely recognized: the DSM-5 (Diagnostic and Statistical Manual of Mental Disorders, Fifth Edition) [11], the NIA-AA criteria (National Institute on Aging and the Alzheimer’s Association) [12], and the ICD-11 (International Classification of Diseases, Eleventh Revision) [13].

Biomarkers are crucial for improving dementia diagnosis and monitoring. Structural Magnetic Resonance Imaging (MRI) reveals brain atrophy, Fluorodeoxyglucose Positron Emission Tomography (FDG-PET) evaluates neuronal activity, and amyloid Positron Emission Tomography (PET) detects amyloid-β plaques characteristic of Alzheimer’s disease. Cerebrospinal fluid biomarkers—reduced amyloid-β42 and elevated total and phosphorylated tau—form the basis of the AT(N) framework, which classifies amyloid pathology (A), tau pathology (T), and neurodegeneration (N) [14,15,16]. Blood-based biomarkers, such as plasma phosphorylated tau (p-Tau181, p-Tau217), neurofilament light chain (NfL), and the amyloid-β42/40 ratio, are gaining attention due to their non-invasive nature and diagnostic potential. Additionally, genetic markers like the APOE ε4 allele serve as risk indicators for Alzheimer’s [17,18]. Oxidative stress biomarkers—including MDA, GSH, SOD, and CAT—are increasingly used in both clinical and preclinical studies to monitor redox imbalance, a contributor to neurodegeneration in dementia [19].

The use of plant-based compounds in therapeutic interventions, known as Phytotherapy, has increasingly been explored for the treatment of neurodegenerative conditions such as dementia. One such compound, curcumin, a polyphenol derived from Curcuma longa, has demonstrated notable neuroprotective potential, largely due to its antioxidant, anti-inflammatory, and anti-amyloidogenic activities [20,21]. In in-vivo models of dementia, including those induced by streptozotocin, scopolamine, and amyloid-β peptides, oxidative stress is a well-established pathological feature.

The assessment of oxidative stress markers in these models provides key insights into the biochemical mechanisms underlying cognitive impairment, particularly by highlighting the role of oxidative damage in neuronal dysfunction. Across multiple studies, elevated levels of markers such as malondialdehyde (MDA) and decreased antioxidant enzyme activity (e.g., superoxide dismutase [SOD], catalase [CAT], and glutathione [GSH]) were consistently associated with impaired memory and learning behaviors. These oxidative stress parameters serve not only as biomarkers of neuronal injury but also highlight the mechanistic pathways through which cognitive impairment can occur, particularly under conditions of neurotoxicity or therapeutic intervention For example, in arsenic-induced neurotoxicity models, oxidative stress has been shown to impair mitochondrial function by reducing cytochrome c oxidase activity and disrupting mitochondrial membrane potential, leading to neuronal energy failure and cognitive decline [22]. Additionally, arsenic exposure activates the redox-sensitive NF-κB pathway, promoting neuroinflammation through elevated TNF-α and IL-1β levels, further contributing to synaptic damage and memory deficits [23,24]. These patterns suggest that oxidative stress is not merely a correlate but a key mechanistic contributor to cognitive impairment. Understanding these pathways provides a mechanistic basis for evaluating antioxidant compounds like curcumin, which are thought to exert neuroprotective effects by restoring antioxidative function and inhibiting neuroinflammatory signalling in preclinical models.

As precision medicine advances, the integration of biochemical markers, particularly oxidative stress biomarkers, into the diagnosis and monitoring of dementia is becoming increasingly relevant. These markers not only facilitate early detection and disease progression tracking but also serve as valuable endpoints in evaluating treatment responses. Given the central role of oxidative stress in dementia pathogenesis, antioxidant-based interventions, including dietary and lifestyle modifications, are gaining traction as potential preventive and therapeutic strategies. Curcumin, due to its multifunctional bioactivity and natural origin, represents a compelling nutraceutical candidate in this context.

Therefore, this systematic review aims to critically evaluate the impact of curcumin on oxidative stress markers in in-vivo models of dementia. By synthesizing available evidence, this review seeks to elucidate the mechanistic basis of curcumin’s neuroprotective effects and inform future treatment approaches, especially in resource-limited settings where affordable interventions are urgently needed.

## 2. Methods

**Figure 1:**
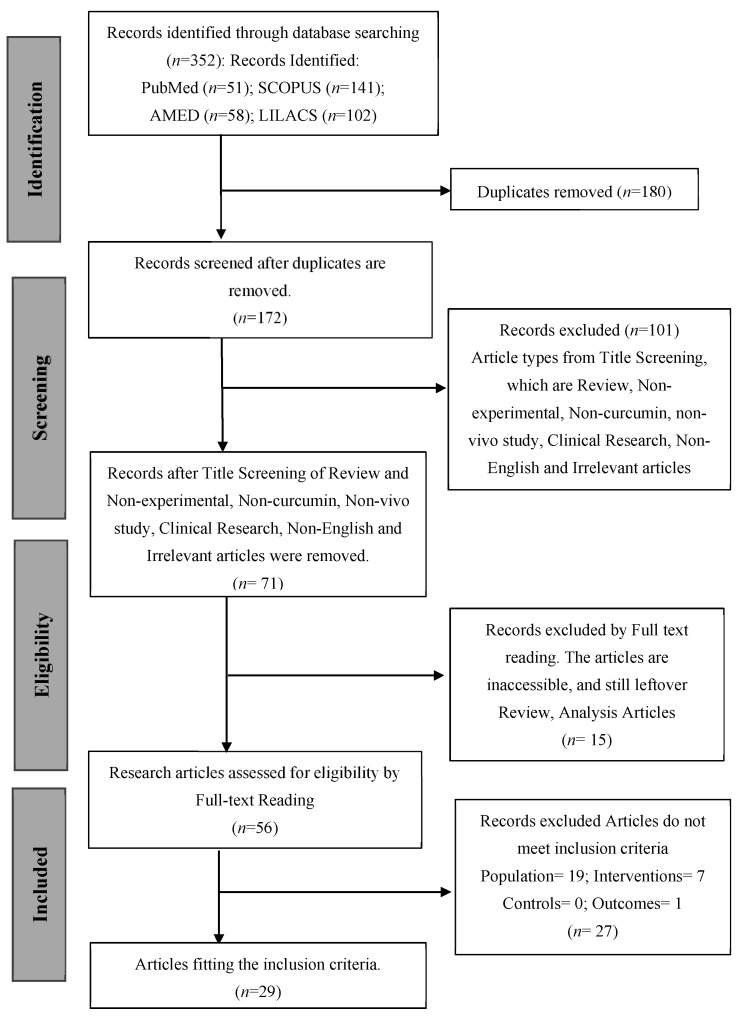
PRISMA flow diagram of study selection.

This systematic review was carried out following the Preferred Reporting Items for Systematic Reviews and Meta-Analyses (PRISMA) guidelines for systematic reviews and meta-analyses [25]. The PRISMA Protocols checklist is presented in Figure 1. Two researchers independently worked at every step of the systematic review (study search and selection, data extraction, and risk of bias assessment). The planned, systematic review was registered on the International Platform of Registered Systematic Review and Meta-analysis Protocols (INPLASY^®^) register (INPLASY202550002; 10.37766/inplasy2025.5.0002).

### 2.1. Search Strategy

Descriptors indexed in Health Science Descriptors (DeCS) and Medical Subject Headings (MeSH) were utilized for the search strategy. The search terms included: Curcumin, Curcuma, in vivo, dementia, oxidative stress, neurodegenerative disease, frontotemporal dementia, neurodegeneration, neuroinflammation, mice, rats, synaptic, cognitive impairment, cognitive abilities, cognitive function, synaptic dysfunction, synaptopathies, and neuropsychological functions, with the search conducted in English. The Boolean operators “AND” and “OR” were used to combine the descriptors. Both MeSH terms and keyword variations were used, and articles were limited to “rodents” and the English language, where possible. The search strategy can be found in Appendix A (Table A1).

The review was limited to papers published on original and experimental studies in rats and mice that examined the antioxidative, anti-inflammatory, and cognitive enhancing properties of curcumin/curcuma. Furthermore, the reference lists of the included papers were scrutinized to identify additional pertinent studies that could be included in this review. In studies with more than one intervention, data for curcumin/curcumin extract-treated groups and corresponding control groups were utilized in this systematic review. All the articles retrieved through these searches were transferred to Microsoft Excel 19 and duplicates removed. Initial screening was done through title and abstract review, with the removal of unrelated articles that ensued. Afterwards, the remaining articles underwent a critical evaluation by screening the full text. Articles without available full texts or irrelevant were excluded as well.

### 2.2. Eligibility Criteria

All studies included in the review were assessed based on predefined inclusion and exclusion criteria, as outlined in Table 1. These criteria were developed following the PICO framework to guide the systematic selection process.

### 2.3. Information Sources

A comprehensive literature search of electronic databases using US National Library of Medicine and the National Institutes of Health, PubMed (http://www.pubmed.gov, accessed on 4 April 2024), SCOPUS (http://www.scopus.com, accessed on 3 April 2024), Allied and Complementary Medicine Database (AMED), AMED (https://www.ebsco.com/products/research-databases, and Latin American and Caribbean Health Sciences Literature, LILACS (https://lilacs.bvsalud.org/en/, accessed on 3 April 2024). These databases were searched for findings published until April 2024 (See Appendix A Table A1).

### 2.4. Data Extraction and Management

Two independent reviewers (BBL and WPL) screened titles and abstracts to identify eligible studies. In cases of disagreement, a third reviewer (SAK) resolved the conflict by determining whether the study met the inclusion criteria. To minimize subjectivity during data collection and entry, three reviewers (MMS, BBL, and KYP) independently extracted data from the included studies and recorded them in separate databases. Data were abstracted using standardized forms that captured key study characteristics, including the first author’s name, publication year, publication country, sample size, animal gender, age, and strain. Additionally, indices such as type of treatment, induction methods, dose, and duration were also expressed. Physiological indices (oxidative markers, inflammatory markers) and behavioral/cognitive measures (e.g., learning, memory, memory performance, locomotor activity, and anxiety-like behavior), and statistical measures for each outcome (e.g., means and standard deviations) were documented. When effect sizes could not be extracted or calculated from the published data, corresponding authors were contacted via email for additional statistical information. Finally, the databases were cross-checked, and any discrepancies were resolved through discussion among the reviewers. To enhance the results’ visual representation, the data were arranged and depicted with figures, and some were displayed as tables.

### 2.5. Quality Assessment

The overall quality of evidence from the included studies was evaluated using the Collaborative Approach to Meta-Analysis and Review of Animal Data from Experimental Studies (CAMRADES) framework, which aims to enhance the design, execution, and reporting of preclinical studies included in systematic reviews and meta-analyses. This evaluation considered several factors, including the risk of bias within individual studies, directness of evidence, precision of effect estimates, heterogeneity among studies, and the potential for publication bias [26]. Bias, in this context, refers to systematic deviations from the true findings or inferences, which may distort study results.

To assess the risk of bias, a structured checklist developed by the Systematic Review Centre for Laboratory Animal Experimentation (SYRCLE) was employed [27]. This tool, adapted from the Cochrane Collaboration’s Risk of Bias Tool, includes ten items grouped into six core domains: selection bias, performance bias, detection bias, attrition bias, reporting bias, and other sources of bias. Each domain was assessed using one of three responses: “Yes” (indicating low risk of bias), “No” (indicating high risk), or “NC” (not clear, due to insufficient information). A point was assigned for each item judged as “Yes”.

Two reviewers (BBL and SAK) independently assessed each study and rated the risk of bias as “low”, “unclear”, or “high” across the following areas: sequence generation, baseline characteristics, allocation concealment (selection bias), random housing and blinding (performance bias), random outcome assessment and blinding (detection bias), incomplete outcome data (attrition bias), selective outcome reporting (reporting bias), and other potential sources of bias. After completing their assessments, the reviewers compared and discussed their findings. Any discrepancies in scoring were resolved through consultation with a third reviewer (SC) to reach a consensus.

## 3. Results

### 3.1. Study Selection

The study selection process is summarized in the PRISMA flow diagram (Figure 1). A total of 352 records were initially identified through electronic database searches, with contributions from PubMed (*n* = 51), SCOPUS (*n* = 141), AMED (*n* = 58), and LILACS (*n* = 102). After the removal of 180 duplicate records, 172 unique records remained for screening. During the title and abstract screening phase, 101 records were excluded for not meeting the inclusion criteria. These exclusions were primarily due to the articles being reviews, non-experimental studies, unrelated to curcumin, non-in vivo studies, clinical research, non-English publications, or otherwise irrelevant to the topic under investigation.

This left 71 records for further assessment. Of these, 15 articles were excluded after full-text reading due to being inaccessible or identified as reviews or analytical articles. The remaining 56 full-text articles were assessed for eligibility. Following a detailed evaluation, 27 additional articles were excluded for not fulfilling specific inclusion criteria: 19 lacked the appropriate population, 7 did not meet intervention criteria, 1 lacked outcome data, and none were excluded based on control criteria.

In total, 29 studies [28,29,30,31,32,33,34,35,36,37,38,39,40,41,42,43,44,45,46,47,48,49,50,51,52,53,54,55,56] fulfilled all the predefined inclusion criteria and were incorporated into the final systematic review. For these studies, statistical data were retrieved either directly from the published manuscripts or by contacting the corresponding authors via email. However, for certain studies, the required data could not be obtained due to non-responsiveness from the authors. As a result, those studies lacking sufficient data for meta-analysis were included solely in the qualitative component of the systematic review. The first included study was published in 2001, and 68.9% of the included studies were published during the recent decade (i.e., 2014–2024), indicating that the protective effects of Curcumin on dementia are of increasing recent interest and gaining reasonable traction.

### 3.2. Study Risk of Bias Assessment

**Figure 2:**
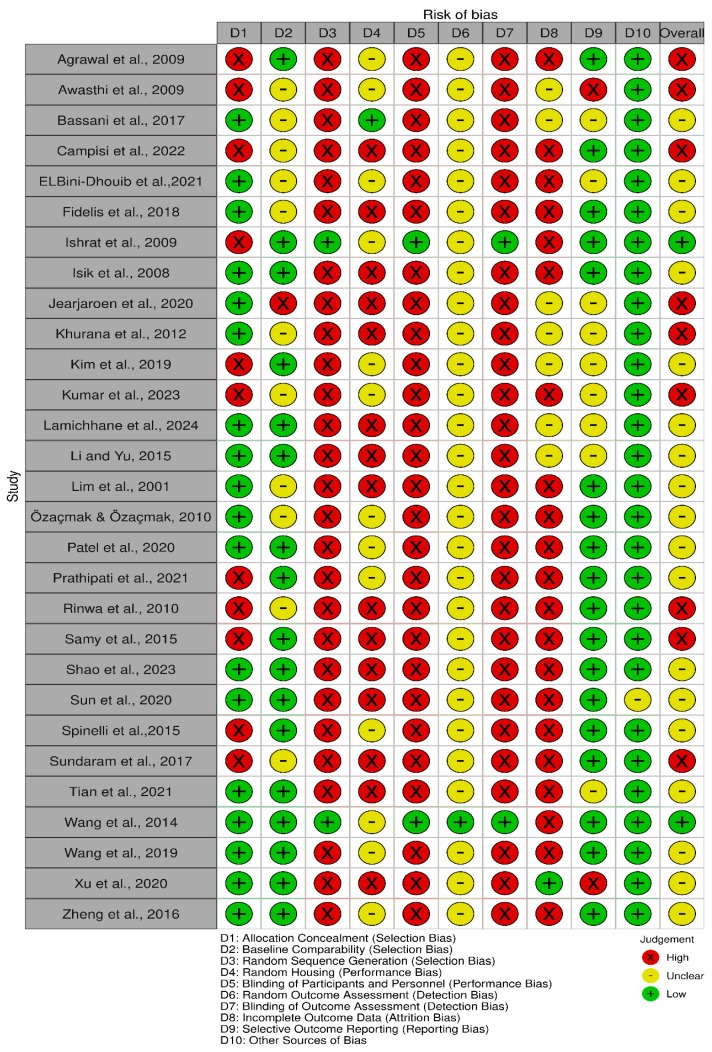
Quality of reporting and bias evaluation conducted with SYRCLE’s risk of bias tool (Risk-of-bias VISualization (robvis) [57]) illustrating the quality of reporting and bias risk in the included studies [28,29,30,31,32,33,34,35,36,37,38,39,40,41,42,43,44,45,46,47,48,49,50,51,52,53,54,55,56].

**Figure 3:**
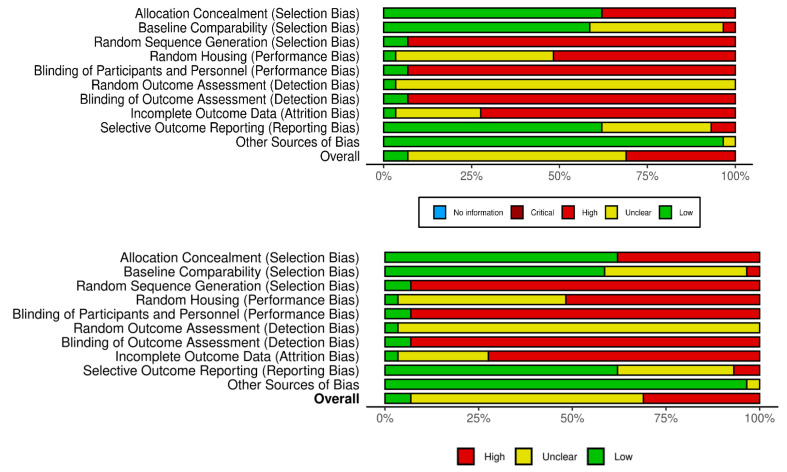
Quality of reporting and bias evaluation conducted with SYRCLE’s risk of bias tool evaluating biases related to selection, performance, detection, attrition, and other factors [57].

The risk of bias in included studies is shown in Figure 2 and Figure 3, in which the quality of reporting and bias evaluation conducted with SYRCLE’s risk of bias tool to evaluate biases related to selection, performance, detection, attrition, and other factors is illustrated [27]. Eighteen (18) studies [30,32,33,35,38,40,41,42,43,44,45,48,49,50,52,54,55,56] had detected unclear biases related to random housing, sequence generation, detection bias and performance bias, Nine (9) studies [28,29,31,36,37,39,46,47,51] had high risk of allocation bias due to probably allocation to the various groups not adequately concealed while two (2) studies [34,53] had low risk of bias.

### 3.3. Study Characteristics

The general characteristics of the included studies are presented in Table 2.

#### 3.3.1. Demographic Data

**Figure 4:**
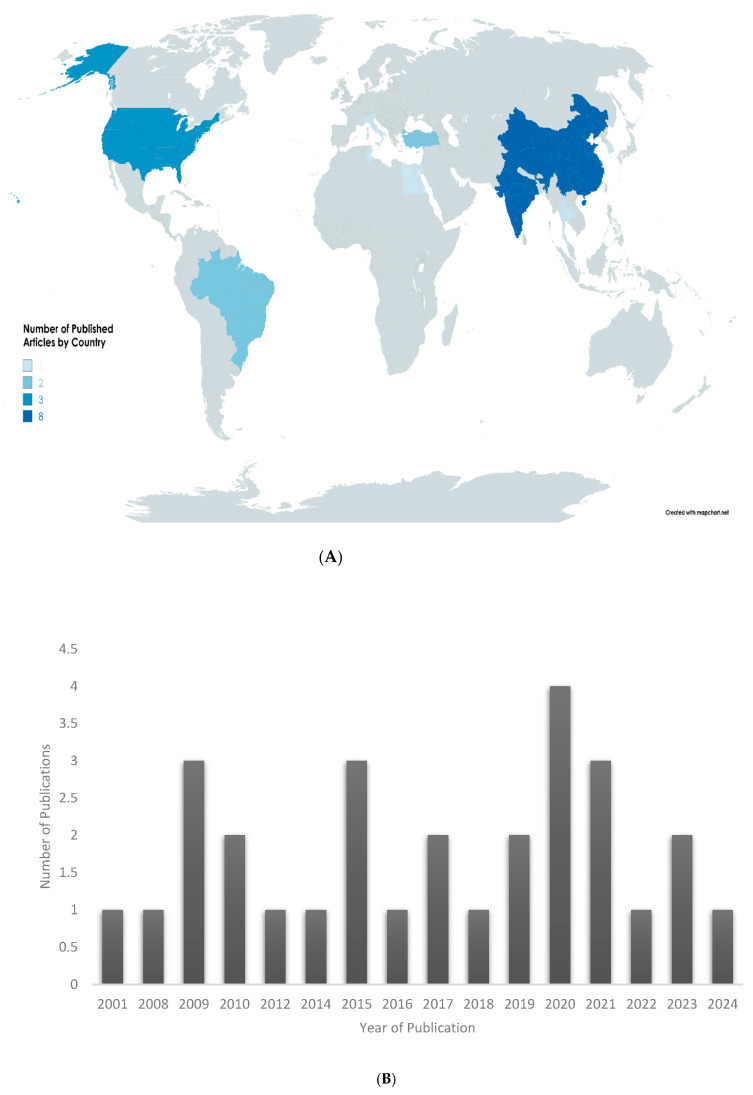
Geographical distribution ((**A**)—upper panel) and publication year ((**B**)—lower panel) of included studies.

The selected studies were conducted by researchers from 11 different countries. Among the countries, China [41,48,50,52,53,54,55,56] and India [28,29,37,39,45,46] are the leading countries with 27.6% each, followed by the United States of America [40,42,51], which has 10.3%, and Brazil [30,33] and Turkey [35,43], each with 6.9%. Italy [31], Korea [38], Singapore [51], Tunisia [32], Egypt [52], and Thailand [36] each have 3.45%, as shown in Figure 4A. The years of the different studies included in this review are depicted in Figure 4B. The earliest study included in the review was published in 2001, with 68.9% of the selected articles published within the last decade (2014–2024), reflecting a growing and sustained research interest in the neuroprotective potential of curcumin in the context of dementia.

#### 3.3.2. Animal Models

**Figure 5:**
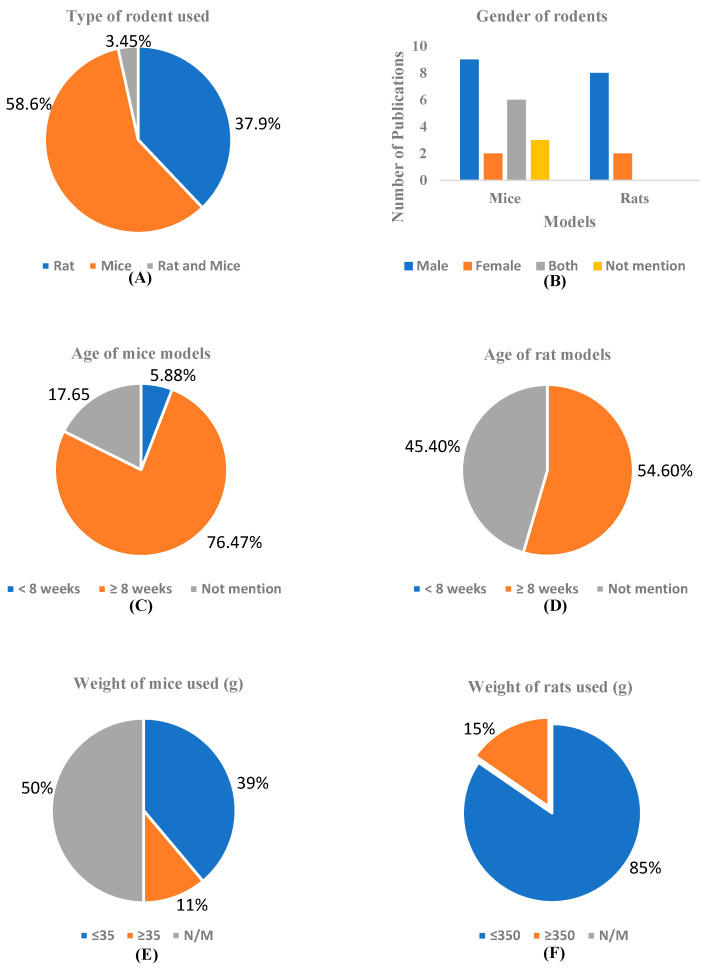
Types of rodents (**A**), gender of rodents (**B**), age of rodents (**C**,**D**), and weight distribution of the rodents (**E**,**F**) used in the included studies reflect the demographics of rodents assessed for the effects of curcumin in research on dementia.

As depicted in Figure 5A, in the in-vivo models of dementia studies under review, 37.9% (*n* = 11) used rats [28,30,32,34,35,37,39,41,43,45,47], 58.6% (*n* = 17) used mice [29,31,33,37,38,40,42,44,46,48,49,50,51,52,53,55,56], while 3.45% (*n* = 1) used both rats and mice [54]. Also, as observed in Figure 5B, a total of 29 publications were reviewed across in-vivo dementia models using mice and rats. For studies using mouse models (*n* = 16), 43.75% used male animals [29,33,36,38,48,50,56] exclusively (*n* = 7), while female-only models [40,44] accounted for 12.5% (*n* = 2). Studies that used both sexes [42,44] represented 12.5 % (*n* = 2), and 18.75% (*n* = 3) did not report on the gender of the animals [31,38,52]. In rat models (*n* = 11), 80% of the studies (*n* = 8) employed male animals [28,30,32,34,35,37,41,47], and 20% (*n* = 2) used females only [39,43].

The ages of the rodents used in the included studies are represented in Figure 5C,D. Among the 17 mouse models analyzed, the majority, 76% (*n* = 13), were reported to be 8 weeks of age or older [29,31,33,40,43,48,49,50,51,52,53,55,56]. A smaller portion, 18% (*n* = 3), did not mention the age of the mice used [36,44,46], while only 6% (*n* = 1) of the studies involved mice that were younger than 8 weeks [38]. These findings indicate a strong preference for using adult mice (≥8 weeks) in dementia-related studies, although a few studies still lack clarity on age reporting. The age intervals “<8 weeks” and “≥8 weeks” for mice were chosen based on their developmental stages, with mice under 8 weeks considered juvenile and those 8 weeks or older regarded as young adults; this distinction ensures greater physiological maturity, reduces variability from ongoing development, and better aligns organ, immune, and hormonal maturity with research objectives, particularly in studies of toxicology, pharmacology, and disease progression.

Also, 54.60% of the rats (6 out of 11) are aged 8 weeks or more [30,34,35,37,41,43]. This indicates that a majority of the rats in the study are above the 8-week threshold. 45.40% of the rats (5 out of 11) have an unspecified age [28,32,39,45,47], suggesting that a significant portion of the subjects lacked age information. There is no study that didn’t mention the age of the rats. The classification of rat age into “<8 weeks” and “≥8 weeks” reflects important developmental milestones, with rats under 8 weeks considered juvenile or peripubertal, experiencing ongoing growth and hormonal changes, while rats 8 weeks or older are regarded as young adults with mature physiology, making them more suitable for toxicological, pharmacological, and disease modeling studies; this distinction aligns with OECD and NIH guidelines to ensure consistency, reduce variability, enhance translational validity, and uphold ethical standards in biomedical research.

Figure 5E,D presents data on the weight distribution of the rodents (mice and rats) used in the included studies. Thirty-nine percent of the mice (7 out of 17) weigh less than 35 g, as indicated in some studies [29,31,43,46,48,54,55]. This indicates that a notable portion of the studies consists of smaller mice, falling into the lightest weight category. Eleven percent of the mice (2 out of 17 studies) weigh 35 g or more [33,36], while 50% of the mice (8 out of 17 studies) lack recorded weight data [38,40,42,49,50,51,52,53]. The division of mouse weight into “≤35 g” and “>35 g” is based on biological development and research practices, where mice weighing ≤35 g are typically young or mid-aged adults with mature but non-aged physiology, ensuring experimental consistency. In contrast, those > 35 g may represent older adults, obese models, or larger genetically modified strains with distinct metabolic or disease profiles. This 35 g threshold aligns with average adult weights for common laboratory strains and aids in accurately interpreting pharmacokinetic, toxicological, and physiological outcomes. Also, 85% of the studies (9 out of 11) used rats that weigh 350 g or less [28,30,32,35,37,39,43,45,47], indicating that the majority of the studies employed rats in the lower weight category. In contrast, only 15% of the studies (2 out of 11) used rats that weigh more than 350 g [34,41]. The selection of the weight intervals ≤350 g and >350 g for rats is based on developmental and physiological stages, with rats ≤350 g typically being younger or adolescent, possessing higher metabolic rates and distinct hormonal, behavioral, and immune profiles. This division helps control for age-related differences, ensures consistency within groups, and allows for clearer analysis of how body weight, and by extension, age or maturity, impacts experimental outcomes.

#### 3.3.3. Induction Method of Dementia in Animal Models

**Figure 6:**
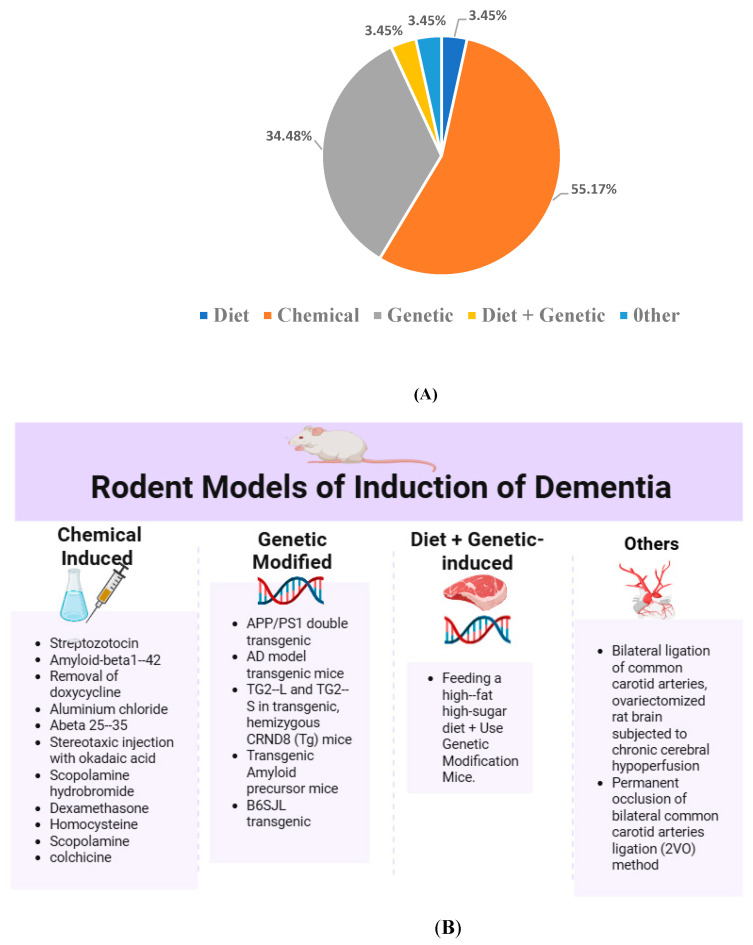
The detailed methods for inducing dementia in these rodents (**A**,**B**) are included in the studies.

In the reviewed studies, five primary approaches to dementia induction were identified: chemical, genetic, dietary, combined diet and genetic, and others (Figure 6 and Appendix A Table A2). These methods reflect the multifactorial nature of dementia and the efforts to accurately model its pathophysiology in an experimental setting.

- Chemical Induction of Dementia Models

Chemical induction was the most frequently employed method for modeling dementia, accounting for 55.17% of all included studies. This approach involves the administration of neurotoxic agents to replicate key neuropathological features of dementia, such as oxidative stress, cholinergic dysfunction, insulin resistance, and amyloid plaque formation. These models are favored due to their cost-effectiveness, ease of administration, and relatively rapid onset of cognitive deficits, making them both reliable and reproducible. Among the chemical inducers, Streptozotocin (STZ) was the most commonly used agent. It featured prominently in several studies [28,29,30,34,35,44,46,47] administered either intracerebroventricularly or systemically to induce insulin resistance in the brain, thereby mimicking sporadic Alzheimer’s disease.

Other frequently used chemical agents include: Scopolamine, a muscarinic receptor antagonist employed to model cholinergic dysfunction [39,44]; Amyloid-beta peptides (A-beta 25–35), representing amyloidogenic insults [34,48]; Dexamethasone, used to simulate glucocorticoid-induced cognitive impairment [36]; Colchicine, which disrupts microtubule integrity, inducing neuronal damage [37]; Aluminum chloride, employed to model neurotoxicity [32]; Homocysteine, implicated in vascular dementia [45]; Okadaic acid, a phosphatase inhibitor that induces tau hyperphosphorylation [53]. Collectively, these agents offer diverse and mechanistically relevant models for studying cognitive impairment and dementia pathology.

- Genetic Induction

Several studies (34.48%) employed transgenic mouse models to investigate genetically predisposed forms of dementia. APP/PS1 and other double or triple transgenic mice [42,49,50,52,53,55,56] are commonly used to recapitulate amyloid plaque deposition and neurofibrillary tangle formation seen in Alzheimer’s disease. TG2-L and TG2-S hemizygous CRND8 mice [31], and B6SJL transgenics [38], further enrich the genetic models used, while p25/Cdk5 hyperactivation [51], induced by removal of doxycycline in genetically modified mice, highlights another mechanism involving tauopathy

- Dietary Induction

Only 3.45% of the studies utilized dietary interventions to induce dementia. Diet-induced cognitive impairment was observed in the study by Lamichhane et al. [40], which involved feeding a high-fat, high-sugar diet, simulating metabolic risk factors associated with dementia. Although this approach is less common, it provides an important link between lifestyle factors and dementia risk.

- Combined Diet and Genetic Induction

A further 3.45% of the studies employed a combined dietary and genetic approach. The approach of Lamichhane et al. [40] models aims to replicate the complex interplay between genetic predisposition and environmental factors in the development of dementia. Although underutilized, this combined method offers a more holistic model of disease development.

- Surgical and Other Models

Another 3.45% represent studies in which surgical interventions and other physiological manipulations were also employed: 2-vessel occlusion (2VO) [39], and bilateral ligation of the common carotid arteries [43], a model of chronic cerebral hypoperfusion, which is relevant in vascular dementia. Also, ovariectomized rats subjected to chronic cerebral hypoperfusion [44] simulate post-menopausal cognitive decline. While these methods are not commonly employed, they may offer unique insights into specific aspects of dementia pathogenesis.

### 3.4. Intervention Characteristics

**Figure 7:**
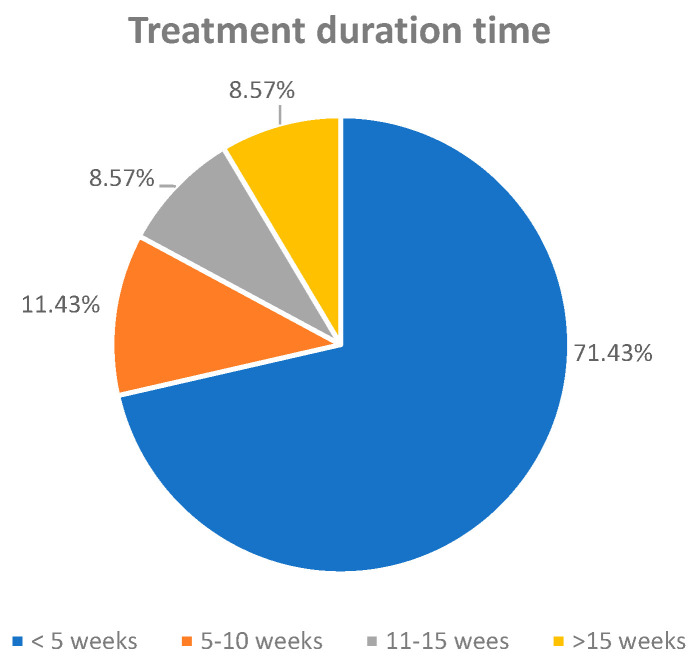
Treatment duration of curcumin/Curcuma in the rodents.

With reference to Figure 7 and Appendix A Table A3, the intervention characteristics are summarized as follows.

#### 3.4.1. Type of Intervention

A total of 29 eligible studies employed curcumin or its derivatives to evaluate its medicinal benefits in animal models of dementia. The majority of the interventions [28,29,30,32,34,35,37,39,54,56,57] involved pure curcumin (86.2%, *n* = 25), while curcumin derivatives or formulations, such as solid lipid nanoparticles, theracurmin, hexahydrocurcumin, and bisdemethoxycurcumin, accounted for 13.8% (*n* = 4) of the studies [31,33,36,55]. Curcumin-loaded nanoparticles [31,33] demonstrated stronger antioxidant responses, while Theracurmin and hexahydrocurcumin showed efficacy at lower doses than standard curcumin. However, combination therapies enhanced the neuroprotective effect, with synergistic results in models treated with curcumin + erythropoietin or probiotics.

#### 3.4.2. Dosing Strategies

Standard doses of curcumin ranged from 10 mg/kg to 400 mg/kg. Low dietary doses were administered in ppm (e.g., 160–5000 ppm in [39,50]), Nanoparticle and liposomal forms were used at comparatively lower concentrations (e.g., 5 µg/kg for bisdemethoxycurcumin; 10–150 mg/kg for nano-formulations) while the most frequently used dose across studies was 100 mg/kg [30,32,37,38,39,41,43] appearing in 9 studies (31.0%).

#### 3.4.3. Treatment Duration

Duration ranged from 1 week (short-term studies [48,54]) to 24 weeks (long-term dietary interventions [42,52]). An analysis of treatment duration across the included studies revealed that the majority (71.43%) implemented curcumin interventions lasting fewer than 5 weeks [28,29,30,31,33,34,35,36,37,39,41,43,44,45,46,48,54,55], reflecting a preference for short-term protocols in preclinical dementia models. A smaller proportion of studies [32,56] employed intermediate durations ranging from 5 to 10 weeks (11.43%), while longer-term interventions extending from 11 to 15 weeks and beyond 15 weeks were each represented by 8.57% of the studies [38,40,42,47,49,52,53]. This distribution highlights a research trend favoring rapid, cost-effective assessments of cognitive and biochemical outcomes, though longer durations may better reflect the chronic progression of dementia in clinical settings.

#### 3.4.4. Treatment Regimens

Daily oral administration was the predominant route (72.4%), either through gavage or dietary incorporation. Alternative day regimens or pre-treatment protocols (before disease induction) were noted in 6 studies (20.7%) [28,29,32,33,37,46]. Combined or adjunct therapies (e.g., curcumin + erythropoietin, curcumin + CoQ10, or curcumin + *Lactobacillus rhamnosus*) appeared in three studies [36,41,44], accounting for 10.3%.

### 3.5. Antioxidative Effects of Curcumin/Curcuma Longa in Dementia Models

**Figure 8:**
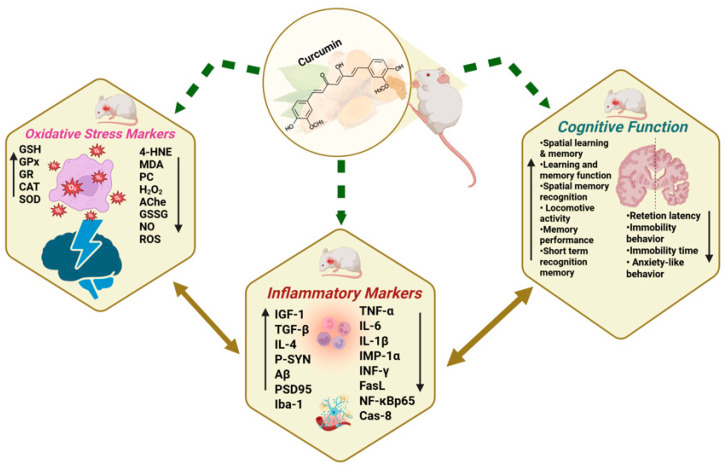
Beneficial effects of curcumin on cognitive functions, inflammatory markers, and oxidative stress.

Aβ peptide accumulation can lead to neuronal damage by increasing reactive oxygen species (ROS) production [19]. Curcumin and its components have antioxidant properties, working by scavenging ROS and reactive nitrogen species (RNS), boosting antioxidant levels, activating Nrf2 signaling, and inhibiting pro-oxidant enzymes [57]. This systematic review of 29 preclinical dementia studies in rodents (see Figure 8 and Table 3) found that only 10 studies reported antioxidant effects of curcumin, shown by reduced oxidative damage markers and enhanced endogenous antioxidant defenses.

Malondialdehyde (MDA), a widely recognized marker of lipid peroxidation, was reduced in all 10 studies (100%) that assessed it (e.g., [28,29,32,34,37,38,43,44,45,47]), representing 34.48% of the total studies reviewed. Similarly, reduced glutathione (GSH), an important non-enzymatic antioxidant, was increased in all 10 studies (34.48%), with no reports of decreases (e.g., [28,29,34,37,38,44,45,46,47,55]), indicating enhanced cellular redox balance. Superoxide dismutase (SOD) activity, a critical enzymatic antioxidant defense, was elevated in 8 of 8 studies (100%) (e.g., [32,33,38,39,44,45,48,55]), further confirming curcumin’s capacity to mitigate oxidative stress. Catalase (CAT) activity increased in 3 out of 4 studies (13.79%), aiding the breakdown of hydrogen peroxide into water and oxygen [33,44,45].

Acetylcholinesterase (AChE) activity, a marker often linked to oxidative damage and cognitive dysfunction, was decreased in seven of nine studies (77.78%)—[28,29,39,44,45,46], corresponding to 24.14% of the total studies, while slight increases in two studies may reflect model-specific variations. Other oxidative markers such as protein carbonyl (PC) and 4-hydroxynonenal (4-HNE) were reduced in three studies (approximately 11%)—(e.g., [34,35,42]), and activities of GPx, GR, and Na^+^/K^+^-ATPase were significantly improved in 7–11% of studies [34,44].

Overall, approximately 85% of the studies reviewed reported positive modulation of at least one oxidative stress parameter following curcumin administration. These findings support curcumin’s potential in mitigating oxidative stress associated with neurodegenerative conditions by decreasing oxidative biomarkers and enhancing antioxidant systems.

### 3.6. Protective Effects of Curcumin/Curcuma Longa on Neuroinflammation in Dementia Models

Neuroinflammation is characterized by the activation of glial cells, primarily microglia and astrocytes, which release pro-inflammatory cytokines (e.g., TNF-α, IL-1β, IL-6) and ROS. While initially protective, chronic glial activation disrupts neuronal homeostasis, exacerbates oxidative stress, and accelerates amyloid-β deposition and tau hyperphosphorylation, ultimately contributing to synaptic dysfunction and neuronal loss. Persistent neuroinflammatory responses further compromise blood-brain barrier integrity, amplifying the infiltration of peripheral immune cells and perpetuating a harmful cycle of inflammation and neurodegeneration. Inflammatory markers were reported in 10 of the 29 studies (see Figure 8 and Table 3). A notable trend was a consistent reduction in pro-inflammatory cytokines and markers, with some studies also reporting increases in anti-inflammatory cytokines.

Reductions in pro-inflammatory markers were consistently reported across several studies. Specifically, interleukin-6 (IL-6) levels were found to be reduced in two studies [35,38]. Interleukin-1 beta (IL-1β) showed a decrease in three studies [42,48,51], while TNF-α levels were also reduced in three studies [39,48,51]. A decrease in interferon-gamma (INF-γ) was observed in one study [32]. Additionally, NF-κB-p65, a critical transcription factor involved in the inflammatory response, was reduced in one study [35]. MIP-1α was also found to be decreased in one study [51]. In terms of markers associated with apoptotic pathways, Fas ligand (FasL) and Caspase-8 levels were reduced in one study [49]. Furthermore, a reduction in ionized calcium-binding adaptor molecule 1 (Iba-1), a marker of microglial activation, was reported in one study [38]. Out of the 10 studies that reported inflammatory outcomes, 80.0% (8/10) observed reductions in at least one pro-inflammatory marker.

Increases in anti-inflammatory markers were also reported in the reviewed studies. Interleukin-4 (IL-4) was found to be increased in one study [32]. Insulin-like growth factor 1 (IGF-1), known for its neuroprotective and anti-inflammatory properties, was elevated in one study as well [36]. Additionally, an increase in phosphorylated synapsin (P-SYN) was observed in one study [49], which indirectly suggests a reduction in neuroinflammation. Overall, 40.0% (4 out of 10) of the studies that assessed inflammatory markers reported an increase in anti-inflammatory cytokines or neuroprotective signals.

Overall, among the studies that assessed inflammatory markers, a majority (over 80%) reported a reduction in pro-inflammatory cytokines following curcumin intervention. A smaller proportion (about one-fourth) also reported an increase in anti-inflammatory or neuroprotective markers. These findings support curcumin’s role in mitigating neuroinflammation, which may contribute to improved cognitive function in dementia models.

### 3.7. Ameliorative Effects of Curcumin/Curcuma Longa on Cognitive Functions in Dementia Models

- Cognitive Function Improvements

Several studies report improvements in various aspects of cognitive function following curcumin administration. Improvements in spatial learning and memory are particularly prominent, with approximately 15 out of 27 studies (51.7%), including those by Agrawal et al. [28], Awasthi et al. [29], Ishrat et al. [34], and Wang et al. [54], reporting notable enhancements (see Figure 8 and Table 3). These improvements are typically indicated by a reduction in escape or transfer latency and an increase in the percentage of correct alternations during behavioral testing.

Recognition memory was reported to improve in four studies (13.3%), such as those conducted by Bassani et al. [30], Kim et al. [38], El Bini-Dhouib et al. [32], and Lamichhane et al. [40]. These studies observed enhancements in short-term memory and recognition tasks following curcumin treatment. Learning and retention performance was enhanced in nine studies (30%), [31,34,37,41,47,48]. Improvements were measured by increased retention latency and better performance in learning and memory tasks, indicating improved cognitive processing.

Reduction in cognitive impairment was reported in seven studies (23.3%), with notable examples from Isik et al. [35], Jearjaroen et al. [36], Khurana et al. [37], and Shao et al. [48]. These studies documented significant declines in cognitive dysfunction indicators post-treatment, demonstrating curcumin’s potential in mitigating cognitive decline.

- Behavioral Improvements Related to Cognition

Behavioral improvements associated with cognitive function were also observed following curcumin administration. A reduction in anxiety-like behavior and immobility behaviors was demonstrated explicitly in four studies [30,32,39,51], accounting for roughly 15% of the total studies reviewed (see Figure 8 and Table 3). Reductions in anxiety and immobility were positively correlated with cognitive improvement markers, suggesting a broader benefit of curcumin on emotional and motivational domains.

Motor and exploratory activities were enhanced in three studies (10%—[30,33,49]). Improvements in locomotor activity were also recorded in studies [33,40,49], representing about 10% of the reviewed data. Notably, a decrease in immobility time, a behavioral marker associated with depressive-like states, was specifically reported by Fidelis et al. [33], highlighting curcumin’s potential role in enhancing motivational behavior.

Overall, curcumin supplementation resulted in enhanced cognitive performance in approximately 80% of the studies reviewed, with strong evidence supporting improvements in spatial memory and learning. Although behavioral benefits such as reduced anxiety and improved locomotion were reported less frequently (20–30%), these findings collectively support the neuroprotective potential of curcumin in addressing dementia-related cognitive and behavioral dysfunction.

## 4. Discussion

In recent years, there has been growing scientific traction in the use of bioactive natural compounds for both the prevention and treatment of neurodegenerative disorders such as dementia. Curcumin, a key polyphenolic compound extracted from Curcuma longa, has emerged as a promising bioactive compound due to its capacity to influence multiple disease-related mechanisms [58]. Based on the widely recognized amyloid hypothesis, the pathological accumulation of amyloid-beta (Aβ) peptides in the brain contributes to the development of toxic soluble oligomers and amyloid plaques. This process, alongside tau protein hyperphosphorylation and the formation of neurofibrillary tangles (NFTs), triggers oxidative stress and neuroinflammation, which are known to be two major contributors to cognitive deterioration [59].

Current clinical interventions for dementia, including cholinesterase inhibitors and the NMDA receptor antagonist memantine, are primarily aimed at alleviating symptoms by modulating specific pathological pathways. However, these pharmacological treatments are often associated with limited therapeutic benefits and potential side effects [60]. As an alternative area of investigation, natural compounds such as curcumin have attracted interest due to their broad-spectrum biological activities, including modulation of oxidative stress, inflammation, amyloid aggregation, and synaptic function, which is sequential to its ability to prevent the onset and slow down the progression of neurogenerative diseases. While the clinical efficacy of such natural products remains under investigation, their multi-targeted mechanisms and lower incidence of adverse effects in preliminary studies suggest potential complementary roles in dementia management.

To deepen our understanding of dementia pathophysiology and potential therapeutic interventions, both transgenic and non-transgenic animal models mimicking key features of dementia have been extensively utilized [61,62]. These models have proven instrumental in evaluating the efficacy of curcumin in mitigating cognitive impairments and neuropathological alterations. A systematic review approach provides a powerful framework to synthesize findings across these preclinical studies, highlighting curcumin’s effectiveness in addressing dementia-related pathology, setting the stage for clinical applications.

In this systematic review, only studies utilizing established dementia-induced animal models were included. The protective effects of curcumin were consistently observed across the included studies. Although the experimental populations and study designs varied, most studies employed similar methods, administering curcumin orally or via injections and comparing its efficacy in chemical-induced, genetically modified, or diet-related models of dementia in mice or rats.

Despite not formally assessing publication bias through funnel plots due to the limited number of studies suitable for such analysis, the majority of outcome comparisons across the 29 studies demonstrated significant beneficial effects of curcumin on cognitive and behavioral functions in relation to antioxidant properties. These included improvements in spatial learning, memory retention, and reductions in anxiety-like behaviors. Within-study effects generally showed consistent trends favoring curcumin treatment; however, instances of statistically insignificant findings were also noted. For example, while Bassani et al. [30] observed improvements in short-term recognition memory, spontaneous locomotion, and anxiety-like behavior, some parameters, such as exploratory behavior, showed minimal or nonsignificant differences between curcumin-treated and control groups (*p* > 0.05). Similarly, Sundaram et al. [51] reported reduced inflammatory markers following curcumin administration, but improvements in learning and memory performance did not reach statistical significance in some behavioral tests. These inconsistencies may reflect variations in experimental models, the pharmacokinetics of curcumin formulations used, and the duration or timing of interventions. Additionally, the complexity of dementia pathology and the multifactorial mechanisms of curcumin’s action (e.g., anti-inflammatory, antioxidant, and anti-amyloidogenic effects) may contribute to differential responses across studies. Overall, despite these limitations, the collective evidence supports curcumin as a promising candidate for ameliorating cognitive deficits associated with dementia.

The pathogenesis of dementia, particularly Alzheimer’s disease (AD), is multifactorial and involves amyloid-beta (Aβ) aggregation, tau protein hyperphosphorylation, oxidative stress, neuroinflammation, apoptosis, neurofibrillary tangle formation, and neuronal death. Numerous studies included in this review reported that curcumin exerts neuroprotective effects across multiple pathological pathways. For instance, Ishrat et al. [34] and Kim et al. [38] demonstrated that curcumin reduced oxidative stress by enhancing antioxidant defenses such as glutathione (GSH), superoxide dismutase (SOD), and catalase (CAT) activities. Additionally, malondialdehyde (MDA), a key lipid peroxidation marker, carbonyl (PC), and 4-hydroxynonenal (4-HNE) were all reduced in the studies reviewed, indicating that curcumin improved cellular antioxidant defenses and reinforced its capacity to enhance endogenous defenses against ROS and RNS. Overall, approximately 85% of the studies demonstrated positive modulation of at least one oxidative stress parameter, corroborating the strong antioxidative potential of curcumin.

Chronic neuroinflammation driven by activated glial cells is another critical driver of neurodegenerative progression. Curcumin consistently attenuated neuroinflammatory markers in rodent dementia models. Among the 10 studies reporting inflammatory outcomes, over 80% observed significant reductions in at least one pro-inflammatory cytokine. Curcumin also showed the ability to inhibit neuroinflammation, evidenced by decreased levels of pro-inflammatory cytokines, including TNF-α, IL-1β, and IL-6, as reported by Shao et al. [48] and Isik et al. [35]. Moreover, curcumin promoted synaptic preservation and neuronal function, as reflected by increases in synaptophysin and PSD-95 expression [38], and improved cholinergic transmission through enhanced acetylcholinesterase (AChe) modulation.

The cognitive benefits of curcumin were prominently observed. Improvements in spatial learning and memory were reported in 51.7% of the studies, demonstrating that curcumin enhances hippocampus-dependent memory processes crucial for navigation and learning. These effects were often indicated by reduced escape and transfer latency times and enhanced spontaneous alternation behaviors in maze-based tasks. Recognition memory improvements were documented in four studies (13.3%), reflecting curcumin’s ability to support short-term and object recognition memory. Enhanced learning and retention performance were noted in nine studies (30%), while reductions in cognitive impairment scores were reported in seven studies (23.3%). Behavioral domains linked to cognition, such as anxiety-like and immobility behaviors, also showed significant improvements. Anxiety-like behavior was reduced in reviewed studies, and enhanced locomotor and exploratory activities were noted. These behavioral improvements complement the cognitive data, suggesting that curcumin also ameliorates emotional and motivational dysfunction associated with dementia.

While this review focuses on rodent models, findings from other species support the conserved neuroprotective effects of curcumin. For instance, non-human primate studies have demonstrated that curcumin crosses the blood-brain barrier and accumulates in brain regions associated with memory and neuroinflammation. In aged and middle-aged rhesus monkeys, curcumin supplementation was associated with reduced amyloid deposition and improved synaptic protein expression in the hippocampus [63,64]. Similarly, in lower model organisms, curcumin has shown cognitive and lifespan-enhancing effects via modulation of conserved pathways. In the Drosophila melanogaster model, Curcumin-supplemented diets improve antioxidant enzymes and alter acetylcholinesterase gene expression level [65,66]. In Caenorhabditis elegans, Cur2004-8, a synthetic curcumin derivative, extends lifespan and modulates age-related physiological changes. The anti-aging effects of Cur2004-8 are linked to the activation of the DAF-16 transcription factor. This activation is crucial for preventing amyloid-beta-induced toxicity and reversing high-glucose-diet-induced mortality [67]. In zebrafish, curcumin improved cognitive performance in scopolamine-induced memory deficits, potentially through cholinergic and antioxidant mechanisms [68]. These findings from non-rodent models reinforce the hypothesis that curcumin’s cognitive benefits may operate through evolutionarily conserved pathways, including oxidative stress mitigation, inflammatory signaling modulation, and mitochondrial preservation.

In summary, in this review, evidence of curcumin’s pleiotropic neuroprotective effects appears to result from its combined antioxidative, anti-inflammatory, and anti-amyloidogenic activities has been established. By scavenging ROS and RNS, enhancing endogenous antioxidant enzymes, downregulating pro-inflammatory cytokines, enhancement of anti-inflammatory cytokines, reduction in amyloid plaque formation and tau hyperphosphorylation, modulation of apoptotic pathways and neuronal survival signals, and improving synaptic protein expression (e.g., synaptophysin, PSD-95), curcumin targets multiple key mechanisms implicated in dementia progression. Moreover, curcumin’s modulation of cholinergic neurotransmission via reductions in AChE activity may directly contribute to its cognitive benefits. These multifaceted actions position curcumin as a promising candidate for multi-targeted treatment approaches in dementia.

### Limitations

While this systematic review provides valuable insights into the potential of curcumin in ameliorating dementia-related cognitive and behavioral deficits, several limitations must be acknowledged. A primary limitation was the relatively small number of studies eligible for meta-analysis. Although many studies initially met the inclusion criteria, most did not report the necessary statistical data (means, standard deviations, or error measures) required for effect size calculations. Improved statistical reporting would enhance the rigor and comparability of preclinical research.

This systematic review only included studies on animal models, and the results may not be generalizable to human populations without clinical trials. Significant variability existed across included studies in terms of animal species, strains, dementia models (e.g., Alzheimer’s disease induced by Aβ peptides, scopolamine, STZ, etc.), curcumin dosages, treatment durations, formulations, administration routes, and treatment durations, which may have introduced heterogeneity affecting the pooled results.

Publication bias remains a concern, as studies with positive findings are more likely to be published than those reporting negative or inconclusive results. Moreover, several studies lacked methodological rigor, including omissions of randomization procedures, blinding, and sample size calculations, raising the risk of bias. Furthermore, curcumin’s well-documented poor bioavailability was not adequately addressed in many studies. Only a few utilized enhanced delivery systems such as nanoparticles or adjuvants, possibly contributing to the variability in treatment efficacy observed.

## 5. Conclusions

Overall, the findings of this systematic review provide compelling preclinical evidence that curcumin has the potential to ameliorate cognitive deficits, reduce neuroinflammation, and improve behavioral outcomes associated with dementia. Nevertheless, high-quality, well-reported preclinical studies and robust clinical trials are crucial for confirming curcumin’s potential as a viable treatment and translating it into clinical practice.

## Figures and Tables

**Table 1:** Inclusion and Exclusion Criteria.

Inclusion Criteria	Exclusion Criteria
Studies in dementia animal models Treatment with Curcumin and its derivatives	Studies conducted in vitro Non-curcumin, clinical research
Articles that were published in English	Non-English, Non-experimental
Controlled study design	Reviews, commentaries and unpublished studies
Studies with one or more of the following: oxidative stress, inflammation and cognitive function in relation to curcumin	Publications without full text access and Studies lacking relevant outcomes

**Table 2:** General characteristics of included studies.

Author	Year	Country	No. of Animals	Characteristics of Rodents Used				
			Total	Per Group	Types of Rodents	Weight (g)	Age (wk.)	Sex (M/F)
Agrawal et al. [28]	2009	India	40	5	Sprague-Dawley SD rats	200–250	N/M	M
Awasthi et al. [29]	2009	India	N/M	6–8	Swiss albino mice	25–30	8	M
Bassani et al. [30]	2017	Brazil	35	6–8	Wistar rats	300–340	12–16	M
Campisi et al. [31]	2022	Italy	20	5	WT & Tg mice	20–30	16	N/M
El Bini-Dhouib et al. [32]	2021	Tunisia	42	6	Wistar albino rats	142–148	N/M	M
Fidelis et al. [33]	2018	Brazil	N/M	7–8	Swiss mice	40–50	12	M
Ishrat et al. [34]	2009	India	40	10	Wistar rats	470–500	52	M
Isik et al. [35]	2008	Turkey	24	8, 7, 8	Wistar rats	225–275	52	M
Jeerajaroe et al. [36]	2020	Thailand	84	12	ICR mice	35–45	N/M	M
Khurana et al. [37]	2012	India	104	8	Wistar rats	250–350	12–14	M
Kim et al. [38]	2019	Korea	N/M	5–10	B6SJL mice	N/M	6–7	M
Kumar et al. [39]	2023	India	42	6	Wistar rats	160–200	N/M	F
Lamichhane et al. [40]	2024	USA	N/M	N/M	3 × Tg-AD mice, B6129SF2/J mice	N/M	28	F
Li and Yu [41]	2015	China	70	N/M	Sprague-Dawley rats	250–300; 400–450	12–24; 72–244	M; M
Lim et al. [42]	2001	USA	28	5–9	APP/PS1 mice	N/M	40	M, F
Özçamak & Özçamak [43]	2010	Turkey	30	10	Wistar rats	200–250	16–24	F
Patel et al. [44]	2020	India	30	6	Swiss albino mice	25–30	N/M	F
Prathipati et al. [45]	2021	India	60	10	Sprague-Dawley rats	230–270	N/M	M
Rinwa et al. [46]	2010	India	80	10	Swiss mice	20–30	N/M	M, F
Samy et al. [47]	2015	Egypt	32	8	Wistar rats	220–250	N/M	M
Shao et al. [48]	2023	China	30	10	C57BL/6J mice	25–30	8	M
Spinelli et al. [49]	2015	USA	7	4, 3	Offspring of Syn-GFP male mice and BDF1 female mice	N/M	12	M, F
Sun et al. [50]	2020	China	15	5	APP/PS1 mice	N/M	24	M
Sundaram et al. [51]	2017	Singapore	N/M	3	Offspring mice (p25Tg mice)	N/M	24	M, F
Tian et al. [52]	2021	China	15	5	APP/PS1, C57BL/6J mice	N/M	24	N/M
Wang et al. [53]	2014	China	33	11	APP/PS1 Double transgenic mice	N/M	24	M, F
Wang et al. [54]	2019	China	6	3	Sprague-Dawley rats	188–218	7–8	M
Xu et al. [55]	2020	China	40	10	C57BL/6 mice	N/M	6–8	M
Zheng et al. [56]	2016	China	N/M	≤5	Offspring mice (5 × FAD mice)	N/M	16	M

**Table 3:** The summary of outcomes related to parameters associated with cognitive function, inflammation, and oxidative stress highlights the effects of curcumin in dementia-induced rodent models.

Studies	Oxidative Stress Markers	Inflammatory Markers	Cognitive Function Parameters
Agrawal et al., 2009 [28]	↓ MDA, ↓ AChe, ↑GSH	-	↓ Spatial learning Memory (Escape latency)
Awasthi et al., 2009 [29]	↓ MDA, ↓AChe, ↓ROS, ↓NO↑GSH	-	↓Spatial learning memory (Escape latency) ↑ Memory and learning
Bassani et al., 2017 [30]	-	-	↓Short-term spatial memory, ↓Anxiety-like behavior ↓Spontaneous locomotion and exploratory behavior ↑Short-term recognition memory
Campisi et al., 2022 [31]	-	-	↑Memory performance
ELBini-Dhouib et al., 2021 [32]	↓ MDA, ↑AChe, ↑SOD, ↑CAT	↓INF-γ, ↑ IL-4	↓ Anxiety-like behavior ↓Mark impairment in memory recognition
Fidelis et al., 2018 [33]	↓ CAT ↑SOD, ↑RS, ↑SOD/CAT, NPSH↔	-	↓ Immobility behavior, ↓Immobility time ↑Locomotor activity,
Ishrat et al., 2009 [34]	↓4-HNE/MDA, ↓H_2_O_2,_ ↓PC, ↑GSSG, ↑GSH, ↑GPx, ↑GR, ↑Na^+^-K^+^ ATPase, ↑ChAT	-	↑ Retention latency, ↑Spatial learning and memory,
Isik et al., 2008 [35]	↓4-HNE	↓IL-6, ↓NF-κB-p65	↑Spatial learning and memory, ↓ Cognitive impairment
Jearjaroen et al., 2020 [36]	-	↑IGF-1	↓ Cognitive impairment,
Khurana et al., 2012 [37]	↓MDA, ↓LPO ↑GSH, ↑AChe,	-	↓ Cognitive impairment ↓Retention latency ↑ Step down latency
Kim et al., 2019 [38]	↓ MDA, ↑GSH, ↑SOD	↓Iba-1, ↑Synoptophysin, ↑PSD95,	↑ Recognition memory ↑ Spatial memory
Kumar et al., 2023 [39]	↓ AChe, ↑SOD	↓TNF-α,	↓Transfer latency, ↓Anxiety-like behavior ↑%alterations on memory function,
Lamichhane et al., 2024 [40]	-	-	↑Spatial recognition memory, ↔ locomotive behavior
Li and Yu, 2015 [41]	-	-	↓Escape latency, ↑Learning & memory ability
Lim et al., 2001 [42]	↓ PC,	↓IL-1β	-
Özaçmak & Özaçmak, 2010 [43]	↓ MDA, ↑GSH,	-	-
Patel et al., 2020 [44]	↓MDA, ↑GPx, ↓Ache, ↑SOD, ↑CAT,	-	↓Memory impairment
Prathipati et al., 2021 [45]	↓ MDA, ↓ AChe, ↑GSH, ↑SOD, ↑CAT,	-	↓Transfer latency by spatial memory ↑% Spontaneous alterations
Rinwa et al., 2010 [46]	↓ TBAR, ↓ AChe, ↑GSH	-	↑Spatial learning and memory
Samy et al., 2015 [47]	↓ MDA, ↓TBAR ↑GSH	↓FasL, ↓Cas-8	↑ Spatial, Learning and memory, ↑Retention latency
Shao et al., 2023 [48]	↑SOD	↓TNF-α, ↓IL-6, ↓IL-1β,	↑Learning & memory ability, ↑Spatial working memory,
Spinelli et al., 2015 [49]	-	↑P-SYN,	↑ Motor behavior
Sun et al., 2020 [50]	-	-	↑Spatial learning and memory,
Sundaram et al., 2017 [51]	-	↓TNF-α, ↓IL-1β, ↓MIP-1α ↔ TGF-β,	↓ Learning memory,
Tian et al., 2021 [52]	-	-	↑Hippocampal-dependent spatial learning and memory ability
Wang et al., 2014 [53]	-	-	↑Memory and cognition function,
Wang et al., 2019 [54]	-	-	↑Spatial learning and memory
Xu et al., 2020 [55]	↑GSH, ↑SOD	-	↑Spatial learning and memory, ↑Learning & memory function,
Zheng et al., 2016 [56]	-	-	↑Spatial learning and memory

↑—Increase, ↓—Decrease; ↔—No discernible difference. Interleukin-6 (IL-6); Interleukin-1beta (IL-1β), Tumor necrosis factor-alpha (TNF-α); Interferon-gamma (INF-γ); Myeloperoxidase (MPO); Nuclear factor kappa-light-chain-enhancer of activated B cells (NFκB); Thiobarbituric acid reactive substances (TBARS); 4-Hydroxynonenal (4-HNE); Malonaldehyde level (MDA); Hydrogen Peroxide Content (H202); Protein Carbonyl level (PC); Reduced Glutathione content (GSH); Oxidized Glutathione Level (GSSG); Glutathione Peroxidase activity (GPx); Glutathione Reductase (GR); Sodium/potassium-dependent ATPase activity (Na+-K+ ATPase); Choline acetyltransferase activity (AChe); Superoxide Dismutase activity(SOD); Catalase activity (CAT); Reactive Oxygen species level (RO); Nitric oxide level (NO); Non-protein thiol levels (NPSH).

## Abbreviations

The following abbreviations are used in this manuscript:

IL-6	Interleukin-6
IL-1β	Interleukin-1beta
TNF-α	Tumor necrosis factor-alpha
INF-γ	Interferon-gamma
MPO	Myeloperoxidase
NFκB	Nuclear factor kappa-light-chain-enhancer of activated B cells
TBARS	Thiobarbituric acid reactive substances
4-HNE	4-Hydroxynonenal
MDA	Malonaldehyde level
H_2_O_2_	Hydrogen Peroxide
PC	Protein Carbonyl
GSH	Reduced Glutathione
GSSG	Oxidized Glutathione
GPx	Glutathione Peroxidase
GR	Glutathione Reductase
Na^+^-K^+^ ATPase	Sodium/potassium-dependent ATPase activity
Ache	Choline acetyltransferase
SOD	Superoxide Dismutase
CAT	Catalase
ROS	Reactive Oxygen species
NO	Nitric oxide
NPSH	Non-protein thiol
AD	Alzheimer’s disease
MRI	Magnetic Resonance Imaging
FDG-PET	Fluorodeoxyglucose Positron Emission Tomography
CSF	Cerebrospinal Fluid
Aβ42	Amyloid-beta 42
t-Tau	Total Tau
p-Tau	Phosphorylated Tau
STZ	Streptozotocin
Aβ	Amyloid-beta
MCI	Mild Cognitive Impairment.
AMED	Allied and Complementary Medicine Database
LILACS	Latin American and Caribbean Health Sciences Literature

## Appendix A

**Table A1:** Database search strategy for in-vivo studies investigating the Impact of Curcumin on Oxidative Stress Markers in Models of Dementia.

Database	Keywords	Results
PubMed (4 April 2024)	KP-1: (((curcumin[Title/Abstract]) OR (curcuma[Title/Abstract]))) AND ((“in vivo”[Title/Abstract])) OR (rat[Title/Abstract])) OR (mice[Title/Abstract]))) AND (((dementia[Title/Abstract])) AND ((oxidative stress[Title/Abstract])))	16
	KP-2: (curcumin[Title/Abstract]) OR (curcuma[Title/Abstract]) AND (“in vivo”[Title/Abstract]) OR (rat[Title/Abstract])) OR (mice[Title/Abstract]) AND (dementia[Title/Abstract] OR (frontotemporal dementia[Title/Abstract]) AND (Neurodegeneration[Title/Abstract])	10
	KP-3: (((curcumin[Title/Abstract] OR curcuma[Title/Abstract]) AND (rat[Title/Abstract] OR mice[Title/Abstract] OR “in vivo”[Title/Abstract])) AND (dementia[Title/Abstract])) AND (neurodegenerative disease[Title/Abstract] OR neurodegeneration[Title/Abstract] OR neuroinflammation[Title/Abstract])	16
	KP-4: (((curcumin[Title/Abstract] OR curcuma[Title/Abstract]) AND (rat[Title/Abstract] OR mice[Title/Abstract] OR “in vivo”[Title/Abstract])) AND (dementia[Title/Abstract])) AND (synaptic[Title/Abstract])	4
	KP-5: ((((curcumin[Title/Abstract] OR curcuma[Title/Abstract]) AND (rat[Title/Abstract] OR mice[Title/Abstract] OR “in vivo”[Title/Abstract])) AND (dementia[Title/Abstract]))) AND (cognitive ability[Title/Abstract] OR cognition[Title/Abstract]) (((curcumin[Title/Abstract]) OR (curcuma[Title/Abstract]))) AND ((“in vivo”[Title/Abstract])) OR (rat[Title/Abstract])) OR (mice[Title/Abstract]))) AND ((Cognitive abilities[Title/Abstract])) AND (((dementia[Title/Abstract])))	5
Scopus (3 April 2024)	KS-1: (TITLE-ABS-KEY (curcumin) AND TITLE-ABS-KEY (“in vivo”) OR TITLE-ABS-KEY (rat) OR TITLE-ABS-KEY (mice) AND TITLE-ABS-KEY (dementia) AND TITLE-ABS-KEY (oxidative stress))	66
	KS -2: (TITLE-ABS-KEY (curcumin) AND TITLE-ABS-KEY (“in vivo”) OR TITLE-ABS-KEY (rat) OR TITLE-ABS-KEY (mice) AND TITLE-ABS-KEY (dementia) OR TITLE-ABS-KEY (frontotemporal dementia) AND TITLE-ABS-KEY (oxidative stress))	5
	KS-3: (TITLE-ABS-KEY (curcumin) AND TITLE-ABS-KEY (“in vivo”) OR TITLE-ABS-KEY (rat) OR TITLE-ABS-KEY (mice) AND TITLE-ABS-KEY (dementia) AND TITLE-ABS-KEY (Neurodegenerative disease) OR TITLE-ABS-KEY (Neurodegeneration) OR TITLE-ABS-KEY (Neuroinflammation)	66
	KS-4: (TITLE-ABS-KEY (curcumin) AND TITLE-ABS-KEY (“in vivo”) OR TITLE-ABS-KEY (rat) OR TITLE-ABS-KEY (mice) AND TITLE-ABS-KEY (dementia) AND TITLE-ABS-KEY (synaptic dysfunction) OR TITLE-ABS-KEY ((synaptopathies)	1
	KS-5: (TITLE-ABS-KEY (curcumin) AND TITLE-ABS-KEY (“in vivo”) OR TITLE-ABS-KEY (rat) OR TITLE-ABS-KEY (mice) AND TITLE-ABS-KEY (dementia) AND TITLE-ABS-KEY (Neuropsychological functions) OR TITLE-ABS-KEY (Cognitive abilities)	3
AMED (4 April 2024)	KA-1: TITLE curcumin AND AB dementia AND AB mice or rat AND AB oxidative stress	22
	KA-2: TITLE curcumin AND AB mice or rat AND AB dementia OR frontotemporal dementia AND AB neurodegeneration	6
	KA-3: TITLE curcumin AND AB mice or rat AND AB dementia AND AB neurodegenerative disease OR neurodegeneration OR neuroinflammation	23
	KA-4: TITLE curcumin AND AB dementia AND AB mice or rat AND AB dementia AND AB synaptic dysfunction OR synaptopathies	1
	KA-5: TITLE curcumin AND AB dementia AND AB mice or rat AND AB dementia AND AB neuropsychological functions or cognitive ability	6
	Allied and Complementary Medicine Database (AMED) EBSCO	
LILACS (3 April 2024)	KL-1: Title, abstract, subject: curcumin and rat or mice and dementia and oxidative stress	30
	KL-2: Title, abstract, subject: curcumin and rat or mice and dementia or frontotemporal dementia and neurodegeneration	12
	KL-3: Title, abstract, subject: curcumin and rat or mice and dementia and neurodegenerative disease or neurodegeneration or neuroinflammation	34
	KL-4: Title, abstract, subject: curcumin and rat or mice and dementia and synaptic dysfunction or synaptopathies	16
	KL-5: Title, abstract, subject: curcumin and rat or mice and dementia and cognitive ability	10
	LILACS, scientific health information from Latin America and the Caribbean countries	

**Table A2:** Strategies for inducing dementia in animal models for included studies.

Author, Year	Methods Used	The Construction of the Induction			
		Diet	Chemical	Genetic	Other
Agrawal et al., 2009 [28]	Streptozotocin (STZ)-induced	-	√	-	-
Awasthi et al., 2009 [29]	Streptozotocin (STZ)-induced	-	√	-	-
Bassani et al., 2017 [30]	Streptozotocin (STZ)-induced	-	√	-	-
Campisi et al., 2022 [31]	TG2-L and TG2-S in transgenic,	-	-	√	-
	hemizygous, CRND8 (Tg) mice				
ELBini-Dhouib et al., 2021 [32]	Aluminium chloride induced	-	√	-	-
Fidelis et al., 2018 [33]	Abeta 25–35-induced neurotoxicity	-	√	-	-
Ishrat et al., 2009 [34]	Streptozotocin (STZ)-induced	-	√	-	-
Isik et al., 2008 [35]	Streptozotocin (STZ)-induced)	-	√	-	-
Jearjaroen et al., 2020 [36]	Dexamethasone-induced	-	√	-	-
Khurana et al., 2012 [37]	Colchicine-induced	-	√	-	-
Kim et al., 2019 [38]	B6SJL transgenic	-	-	√	-
Kumar et al., 2023 [39]	Scopolamine-induced	-	√	-	-
Lamichhane et al., 2024 [40]	Feeding a high-fat high-sugar diet,	√	-	√	-
	Use Genetic Modification Mice				
Li and Yu, 2015 [41]	Permanent occlusion of bilateral	-	-	-	√
	caroid arteries ligation (2VO) method				
Lim et al., 2001 [42]	Transgenic Amyloid precursor mice-	-	√	-	
Özaçmak & Özaçmak, 2010 [43]	Bilateral ligation of carotid arteries,	-	-	-	√
	ovariectomized rat brain subjected to				
	chronic cerebral hypoperfusion				
Patel et al., 2020 [44]	Scopolamine-induced	-	√	-	-
Prathipati et al., 2021 [45]	Homocysteine (HCY)-induced	-	√	-	-
Rinwa et al., 2010 [46]	Streptozotocin (STZ)-induced	-	√	-	-
Samy et al., 2015 [47]	Streptozotocin (STZ)-induced	-	√	-	-
Shao et al., 2023 [48]	Amyloid-beta1-induced	-	√	-	-
Sun et al., 2020 [50]	APP/PS1 double transgenic	-	-	√	-
Spinelli et al., 2015 [49]	AD transgenic mice	-	-	√	-
Sundaram et al., 2017 [51]	p25/Cdk5 hyperactivation-induced by	-	√	-	-
	removal of doxycycline in water				
Tian et al., 2021 [52]	AD model transgenic mice	-	-	√	-
Wang et al., 2014 [53]	Double Transgenic mice	-	-	√	-
Wang et al., 2019 [54]	Stereotaxic injection with	-	√	-	-
	okadaic acid (OA)				
Xu et al., 2020 [55]	APP/PS mouse	-	-	√	-
Zheng et al., 2016 [56]	AD transgenic mice	-	-	√	-

**Table A3:** Experimental designs and treatment protocols for animal models of dementia used in the eligible studies.

Author, Year	Types of Treatment	Doses of Treatment	Duration (Weeks)
Agrawal et al., 2009 [28]	Curcumin pre-treated	200 mg/kg	Day 1 to Day 14 (Daily)
	Curcumin post treated	200 mg/kg	Day14 to Day20 (Daily)
Awasthi et al., 2009 [29]	Curcumin pre-treated	10 mg/kg	
	Curcumin pre-treated	20 mg/kg	3
	Curcumin pre-treated	50 mg/kg	
	Curcumin post-treated	25 mg/kg	1
	Curcumin post-treated	50 mg/kg	
Bassani et al., 2017 [30]	Curcumin	25 mg/kg	
	Curcumin	50 mg/kg	4 (+2 days)
	Curcumin	100 mg/kg	
Campisi et al., 2022 [31]	WT mice with solid lipid nanoparticles-curcumin	150 mg/kg	3
	Tg mice with solid lipid nanoparticles-curcumin	150 mg/kg	
ELBini-Dhouib et al., 2021 [32]	curcumin (dissolved in 1mL of corn oil)	100 mg/kg	9 (+5 days)
		100 mg/kg	Phase 1: 9 (+5 days) + Phase 2: 7 (+4 days)
Fidelis et al., 2018 [33]	NLCC: curcumin-loaded nanocapsules	10 mg/mL	Alternative days; Once every 48 h
	Free curcumin in canola oil	10 mg/mL	day 2, 4, 6, 8, 10, 12
Ishrat et al., 2009 [34]	Curcumin	80 mg/kg	3
	Curcumin	80 mg/kg	3
Isik et al., 2008 [35]	Curcumin in a vehicle using a mixture of 1%sodium carboxy methyl cellulose and 1% Tween-80)	300 mg/kg	1 (+3 days)
Jearjaroen et al., 2020 [36]	Hexahydrocurcumin (HHC)	HHC (40 mg/kg)	4
Khurana et al., 2012 [37]	Curcumin	100 mg/kg	4 weeks (twice daily)
		200 mg/kg	4 weeks (twice daily)
		400 mg/kg	4 weeks (twice daily)
		100 mg/kg	4 weeks; started 7 days before colchicine injection; (twice daily)
		200 mg/kg	4 weeks; started 7 days before colchicine injection; (twice daily)
		400 mg/kg	4 weeks; started 7 days before colchicine injection; (twice daily)
		100 mg/kg	for 1 week before colchicine injection (twice daily)
		200 mg/kg	for 1 week before colchicine injection (twice daily)
		400 mg/kg	for 1 week before colchicine injection (twice daily)
Kim et al., 2019 [38]	Theracurmin	100 mg/kg, p.o. once a day	12
		300 mg/kg, p.o. once a day	
		1000 mg/kg, p.o. once a day	
Kumar et al., 2023 [39]	Curcumin	Curcumin (100 mg/kg)	
		Curcumin (200 mg/kg)	3
	Curcumin and CoQ10	Curcumin (200 mg/kg)	
		and CoQ10 (200 mg/kg)	
Lamichhane et al., 2024 [40]	Curcumin	1. NCD + CUR gp- curcumin-supplemented (4 g/kg) with normal chow diet,	
		2. HFHSD + CUR fed gp-curcumin-supplemented (4 g/kg) with high-fat-high-sugar diet	14
Li and Yu, 2015 [41]	2VO + Curcumin	50 mg/kg	4 (+2 days)
	2VO + Curcumin	100 mg/kg	
Lim et al., 2001 [42]	A low dose of curcumin (mixed with diet)	160 ppm	24
	A high dose of curcumin (mixed with diet)	5000 ppm	
Özaçmak & Özaçmak, 2010 [43]	Curcumin mixing with peanut butter	Curcumin (100 mg/kg)	2
Patel et al., 2020 [44]	Curcumin alone	Curcumin (205 mg/kg)	1 (+3 days)
	Curcumin + *Lactobacillus rhamnosus*	Curcumin (205 mg/kg) + *Lactobacillus rhamnosus* (1 × 106 CFU)	
Prathipati et al., 2021 [45]	Curcumin	25 mg/kg	
	Curcumin	50 mg/kg	2
	Curcumin-Solid Lipid Nano Particle	10 mg/kg	
	Curcumin-Solid Lipid Nano Particle	25 mg/kg	
Rinwa et al., 2010 [46]	Curcumin suspended in 0.5% *w/v* sodium carboxymethyl cellulose	Curcumin (20 mg/kg, p.o.). The administration of curcumin was continued (administered 30 min before) during acquisition trial conducted from days 1 to 4. The animals were administered vehicle (0.5% *w/v* CMC, 10 mL/kg, p.o.) only, given 30 min before retrieval trial conducted on day 5” curcumin (20 mg/kg, p.o.) for 14 days and rest of the procedure was same as described as in the above group.”	2
Samy et al., 2015 [47]	Vehicle + curcumin	80 mg/kg/day	12
	Erythropoietin	500 IU/kg	
	Combined Curcumin + Erythropoietin	N/M	
Shao et al., 2023 [48]	Curcumin (dissolved in 10% polyethylene glycol)	150 mg/kg	1 (+3 days)
Spinelli et al., 2015 [49]	Curcumin premixed in soybean oil prior to incorporation into the diet (dietary supplement grade curcumin)	500 ppm	24
	Dietary supplement grade curcumin	15 mg/kg	2 (+1 days)
Sun et al., 2020 [50]	High dose curcumin	200 mg/kg	12
	Low dose curcumin	50 mg/kg	12
Sundaram et al., 2017 [51]	Curcumin	4 g/kg (0.8 g curcumin/kg)	12
Tian et al., 2021 [52]	Curcumin	Low dose: 0.16 g/kg High dose:1 g/kg	24
Wang et al., 2014 [53]	Low dose curcumin (160 ppm)	160 ppm	24
	High dose curcumin (1000 ppm)	1000 ppm	
Wang et al., 2019 [54]	Curcumine	100 μg/mL	1
	Exo-curcumine	100 μg/mL	
Xu et al., 2020 [55]	Bisdemethoxycurcumin	5 µg/kg	4
Zheng et al., 2016 [56]	Curcumin	150 or 300 mg/kg	7 (+4 days)
